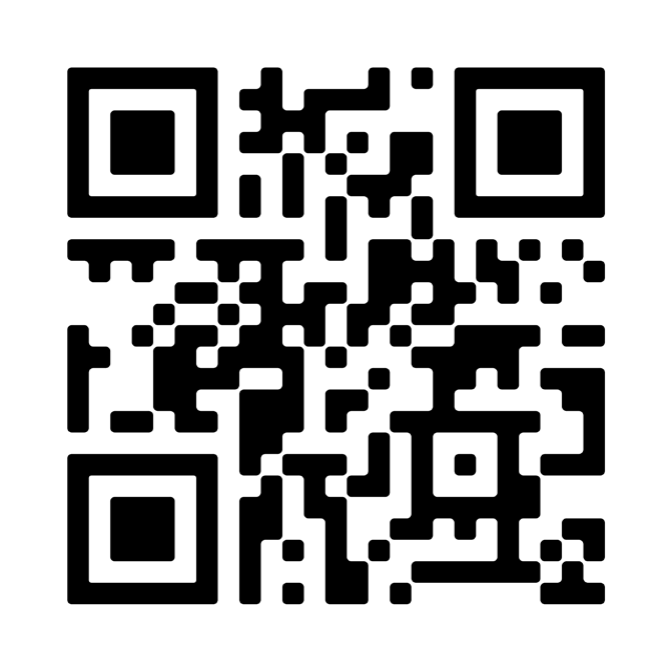# Suture-fixation of a levonorgestrel-releasing intrauterine device under hysteroscopic guidance

**DOI:** 10.52054/FVVO.15.4.107

**Published:** 2023-12-13

**Authors:** P.G. Paul, M Shah, V Sridivya Chowdary, A Anusha Raaj, G Paul

**Affiliations:** Paul’s Hospital, Centre for Advanced Endoscopy and Infertility, Vattekkattu Road, Kaloor, Kochi, Kerala, India

**Keywords:** Adenomyosis, Abnormal uterine bleeding, Hysteroscopy, levonorgestrel-releasing Intrauterine device, Transvaginal ultrasonography

## Abstract

**Background:**

Abnormal uterine bleeding (AUB) is a common gynaecological condition. The levonorgestrel- releasing Intrauterine device (LNG-IUD) is an effective medical treatment. option which carries a small risk of device expulsion. For those who experience expulsion, some may benefit from a more robust surgical approach.

**Objectives:**

To demonstrate the technique for suture fixation of an LNG-IUD under hysteroscopic guidance.

**Materials and Methods:**

Stepwise video demonstration of the technique using a 5mm hysteroscope and a 3mm laparoscopic needle holder. The Institutional Ethical Committee was consulted, and the requirement for approval was waived because the video described a modified surgical technique. Informed consent was obtained from the patient.

**Main outcome measures:**

A 35yr old parous woman with a nine-month history of AUB and severe dysmenorrhoea had an LNG-IUD sited with effective symptom relief. Unfortunately, the device was expelled six months after insertion, and she responded poorly to other medical treatments. Transvaginal ultrasonography (TVUS) suggested posterior wall adenomyosis. Considering her relief of symptoms with the LNG-IUD and history of expulsion, the patient was counselled regarding suture-fixation of the LNG-IUD.

**Results:**

She was followed-up at 6 months post insertion. The LNG-IUD was noted in the uterine cavity without displacement or expulsion.

**Conclusion:**

Hysteroscopy-guided suture fixation of an LNG-IUD is a minimally invasive, effective option for patients with a history of expulsion of an IUD. However, further studies are required to establish the safety and efficacy of this approach.

**Learning Objective:**

To demonstrate LNG -IUD suture fixation technique using hysteroscopy for patients diagnosed with AUB and a history of device expulsion.

## Introduction

Adenomyosis is a common gynaecological condition with symptoms of heavy menstrual bleeding (HMB) and dysmenorrhoea. The diagnosis of adenomyosis is based on transvaginal ultrasonography (TVUS) and magnetic resonance imaging (MRI) (Pontis et al., 2016). Options for treatment of adenomyosis should consider age, reproductive status, and the patient’s clinical symptoms. Hysterectomy is considered the most effective treatment of adenomyosis yet has profound surgical risks as well as rendering the patient infertile (Sharara et al., 2021). The LNG- IUD is beneficial for adenomyosis patients in relieving dysmenorrhoea, heavy bleeding, and uterine volume. The side effects of an LNG-IUD are amenorrhea, spotting and the risk of expulsion (Abbas et al., 2020). Risk factors for IUD expulsion include young age, nulliparity, HMB, previous expulsion, and uterine size > 9cm (Park et al., 2015). The proper placement of the IUD proximate to the uterine fundus also plays an important role in IUD expulsion (Tangtongpet et al., 2017). We demonstrate the technique of hysteroscopy-guided suture fixation of an LNG-IUD in a patient with prior effective symptom control and a history of expulsion.

## Patients and methods

A 35yr old parous woman with a nine-month history of AUB had an LNG-IUD inserted with initial symptom relief yet spontaneously expelled the device six months after insertion. She reattended the department as she had symptom recurrence which was not responding to medical treatment. On evaluation, TVUS highlighted a uterus size of 9.2 x 6.2 cm with posterior wall adenomyosis. Endometrial thickness was 6mm and both ovaries were normal. Considering her relief of symptoms with an LNG-IUD and the device expulsion, she was counselled regarding suture fixation of an LNG-IUD.

## Results

**Figure 1:**
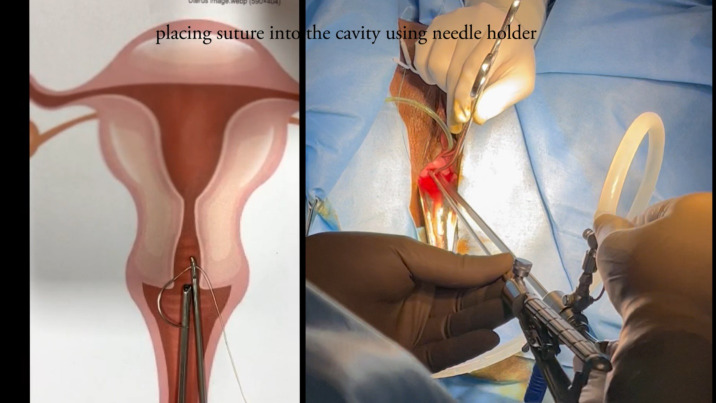
Passing needle.

**Figure 2:**
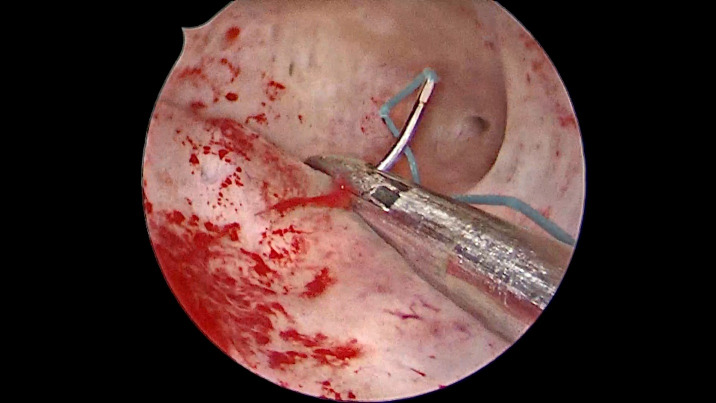
Passing suture.

**Figure 3:**
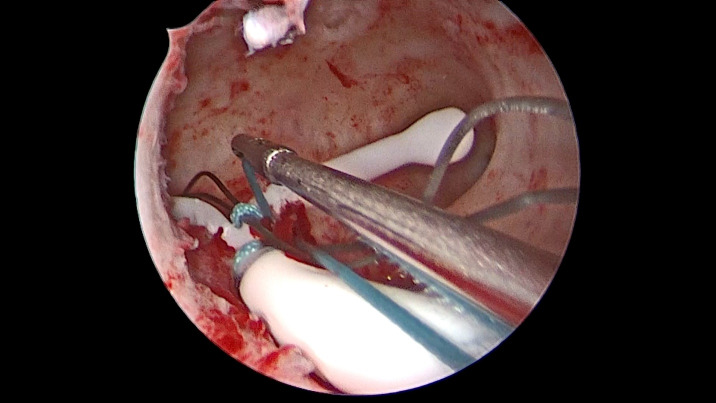
Knot.

Surgery was performed with the 5mm-diameter BETTOCCHI ® hysteroscope (KARL STORZ Tuttlingen, Germany) and demonstrated a normal cervix, uterine cavity, and bilateral ostia. An Ethibond Excel® (Ethicon, Somerville, NJ) 2-0 suture (26mm, ½ circle, Taper cut) was tied to the LNG-IUD (Mirena ® (Bayer AG)) stem and introduced into the uterine cavity using a 3mm laparoscopic needle holder alongside the hysteroscope (Figure 1). The needle was driven over the posterior wall of the uterus with the needle holder held at two thirds along the length of the needle (Figure 2). The needle was taken out of the uterine cavity, simultaneously pulling the LNG-IUD inside the cavity. An extracorporeal knot was made, and the knot was pushed inside using a hysteroscopic grasper to fix the LNG-IUD in place (Figure 3). The patient was discharged 24 hours postoperatively. Follow-up at six months showed the LNG-IUD to be well-sited on TVUS and the patient reported relief of her AUB symptoms.

## Discussion

Conservative management of AUB includes medical treatment and an LNG-IUD for women who decline hysterectomy. 72% of patients using an LNG-IUD showed significant improvement in dysmenorrhea and HMB (Vannuccini et al., 2018). Though an LNG-IUD typically leads to symptomatic relief, it has a risk of expulsion of 8.5% (Cho et al., 2008). These women may need prolonged medical therapy or surgical treatment. As our patient had reported symptomatic relief from HMB with an LNG-IUD prior to device expulsion, we counselled her regarding suture- fixation of the device (Zhang et al., 2022).

We used a 5mm BETTOCCHI® hysteroscope and a 3mm laparoscopic needle holder along the side of the hysteroscope for the LNG-IUD suture fixation. Another way of performing the suture- fixation of an LNG-IUD is by using a special operative hysteroscope - a Hysteroscopic cold knife surgery system (HCSS) - where a needle holder is passed through the operating channel (Zhu et al., 2021). The regular hysteroscope and a 3mm laparoscopic needle holder, which we used, are usually available in most endoscopy operating rooms. Our suturing technique requires a high level of endoscopic skill as the working area is small. We used non-absorbable sutures as suggested by Zhang and colleagues (Zhang et al., 2022). Their group followed up 12 patients after suture-fixation of an LNG-IUD. One had expulsion after 12 months and two patients had a downward shift in the position of the LNG-IUD in the fourth month and sixth month, respectively.

A risk of this intervention includes the needle crossing the uterine wall into the abdominal cavity. This is relatively unlikely given the needle size and thickness of the posterior myometrium in this case. The needle size we used was 26mm, the needle cannot penetrate more than 20mm, and the posterior myometrial wall thickness of our patient was more than 3 cm. There is a possibility of intrauterine adhesions because of the suturing. However, these adhesions can be dealt with during the hysteroscopic removal of the LNG-IUD. Our patient had a follow up at six months without any displacement of an LNG-IUD and good symptom relief.

## Conclusions

Hysteroscopy-guided suture fixation of an LNG- IUD is a minimally invasive and effective option for patients with a history of expulsion of IUD. However, it requires advanced hysteroscopic skills to avoid complications. Further studies are required to establish the safety and efficacy of this approach.

## Video scan (read QR)

https://vimeo.com/844843317/13641d7c54?share=copy

**Figure:**